# Incorporation of trans-rectal color doppler flow imaging and risk-stratification nomogram reduce unnecessary prostate biopsies in suspected prostate cancer patients: a bi-centered retrospective validation study

**DOI:** 10.1186/s12894-023-01245-2

**Published:** 2023-05-03

**Authors:** YiWei Guo, KaiBin Su, MinHua Lu, XiaoPeng Liu

**Affiliations:** 1 Department of Urology, Third Affiliated Hospital, Sun Yat-Sen University, 600 Tianhe Road, Guangzhou, 510630 Guangdong Province China; 2 Department of Urology, Third Affiliated Hospital, Yuedong Hospital, Sun Yat-Sen University, New County Park North Road, Meizhou, Guangdong Province China

**Keywords:** Prostate biopsy, Decision curve analysis, Trans-rectal ultrasonography, Color doppler ultrasonography, Multiparametric magnetic resonance imaging

## Abstract

**Background:**

To explore the role of Trans-rectal Color Doppler Flow Imaging (TR-CDFI) and risk-stratification nomogram in a MRI-directed biopsy pathway and examine its clinical performance, via comparisons between existing four biopsy pathways.

**Methods:**

A Bi-centered retrospective cohort study on biopsy-naïve male population who received ultrasound-guided prostate biopsy from Jan. 2015 to Feb. 2022 was proposed. All enrolled patients should have undergone serum-PSA test, TR-CDFI and multiparametric MRI before biopsy, and subsequently opted for surgical intervention, enabling more accurate pathological grading. We then utilized univariate and multivariate logistic regression analysis to construct a predictive nomogram for risk-stratification. Outcome measurements were overall prostate cancer (PCA) detection rate, clinically significant PCA (csPCA) detection rate, clinically insignificant PCA (cisPCA) detection rate, biopsy avoidance rate and missed csPCA detection rate. Decision curve analysis was used to compare the performances between diagnostic pathways.

**Results:**

Under the criteria mentioned above, 752 patients from two centers were included. Reference pathway (biopsy for all) showed that overall PCA detection rate was 46.1%, csPCA and cisPCA detection rates were 32.3% and 13.8% respectively. Risk-based MRI-directed TR-CDFI pathway, which incorporated both TR-CDFI and risk stratification nomogram, exhibited PCA detection rate of 38.7%, csPCA detection rate of 28.7%, cisPCA detection rate of 7.0%, Biopsy avoidance rate of 42.4%, and missed csPCA detection rate of 3.6%. Decision curve analysis revealed that the risk-based pathway held the most net benefit, under the threshold probability level between 0.1 and 0.5.

**Conclusions:**

The risk-based MRI-directed TR-CDFI pathway out-performed other strategies, balancing csPCA detection and biopsy avoidance. This suggested that incorporation of TR-CDFI and risk-stratification nomogram in the early PCA diagnostic procedures could reduce unnecessary biopsies.

**Supplementary Information:**

The online version contains supplementary material available at 10.1186/s12894-023-01245-2.

## Background

The inclusion of multi-parametric Magnetic Resonance Imaging (mpMRI) in the diagnosis of prostate cancer (PCA), especially before the performance of prostate biopsy on biopsy-naïve males with suspected PCA, has been advocated in the recent European Association of Urology’s guideline on PCA (level of evidence: 1a; strength rating: weak) [1]. Though demonstrating high sensitivity (93%) in the recent PROMIS study [2], mpMRI’s low specificity (41%) on the other hand indicated that it was more likely to produce false positive results that led to unnecessary biopsies, which could further cause multiple adverse effects [3]. Thus, it has been recommended to involve other diagnostic tools such as biomarkers and risk calculators in selecting candidates for mpMRI as well as optimizing the diagnostic strategy for those with negative mpMRI outcomes [1].

The possible hyper-vascularizing nature [4] of potentially malignant prostate lesions had enabled Trans-rectal Color Doppler Flow Imaging (TR-CDFI) to play a more prominent role in the early diagnosis of PCA, though preliminary results suggested only limited improvements over traditional grey-scale ultrasonography [5], and by far its evaluation is still subjective in nature [6]. Nevertheless, with relatively easier accessibility, higher level of specificity [5] (80-87%) and less cost compared to mpMRI, TR-CDFI could still serve as a valuable addition in a mpMRI-directed diagnostic pathway. Based on these considerations, we conducted a retrospective study to examine the effects of TR-CDFI’s integration into existing diagnostic strategies.

## Methods

### Study designs

This retrospective study was approved by the ethic committee of Third Affiliated Hospital of Sun Yat-Sen University and that of Third Affiliated Hospital of Sun Yat-Sen University, Yuedong hospital, with informed consents from all patients involved. All aspects of this research were conducted in accordance with the Declaration of Helsinki. Biopsy-naïve patients who received PI-RADS compliant mpMRI, TR-CDFI with a subjective blood-flow rating scale [6] and other routine examinations prior to ultrasound-guided targeted (TBx) or systematic prostate biopsies (SBx) in two centers were preliminarily included. Then, we further selected those who received surgical interventions (transurethral enucleation of prostate or radical prostatectomy, based on the results of biopsies) and had histo-pathological analyses on their removed prostate tissues, in order to achieve a more accurate pathological classification.

### Patients

752 biopsy-naïve ethnic Chinese patients from the Third Affiliated Hospital of Sun Yat-Sen University (n = 502) and Third Affiliated Hospital of Sun Yat-Sen University, Yuedong hospital (n = 250) who were suspected with PCA and received ultrasound-guided SBx or TBx as well as subsequent surgical intervention, under the timeframe between Jan. 2015 and Feb. 2022, were included. Suspicion of PCA was defined as possessing at least one of the three features: (1) elevated serum PSA level (threshold varied from 2.5ng/mL to 4ng/mL among different urologists in actual clinical practices, and is in accordance to the standards in an existed literature [7]). (2) suspicious rectal examination (cT ≥ 2). (3) family history of PCA. Their detailed baseline characteristics are described in Table 1.

**Table 1:** Detailed baseline characteristics of patients included (n = 752)

Parameters		Statistic	Obs
Age		67 (61,73)	Median, IQR
PSA (ng/mL)		11.89 (7.385,24.3)	Median, IQR
Prostate Volume (mL)		80.92 (56,119)	Median, IQR
PSA density		0.15 (0.08,0.33)	Median, IQR
PI-RADS scores (highest)			
≤ 2		123 (16.4%)	n (%)
3		308 (41.0%)	n (%)
≥ 4		321 (42.6%)	n (%)
mSBRS scores			
< 4		357 (47.5%)	n (%)
≥ 4		395 (52.5%)	n (%)
Number of targeted biopsy targets on MRI			
1		424 (56.4%)	n (%)
2		153 (20.3%)	n (%)
3		152 (20.2%)	n (%)
4		23 (3.1%)	n (%)
Risk stratification based on risk level determined through predictive nomogram (PCA risk ≥ 0.5 or not)			
High (PCA risk ≥ 0.5)		354 (47.1%)	n (%)
Low (PCA risk<0.5)		398 (52.9%)	n (%)

### Assessment of TR-CDFI

The patient was placed in the left lateral decubitus position and the radiologist performed a digital-rectal examination. All patients were then examined using an endo-cavity 5- and 7-MHz probe (Aloka pro-sound 3500, Japan). Radiologists with more than 5 years of experience on prostate ultrasonography first performed the B-mode gray-scale examination, followed by color Doppler sonography. The volumes of prostate were first estimated. Subsequently, all TR-CDFI images were re-assessed and scored by Dr. Lu and Dr. Liu, two urologists with 15 years’ worth experience in prostate ultrasonography, in reference to a modified subjective blood-flow rating scale (mSBRS) based on the work of Mitterberger et.al [6]: score 1: definitely benign, minimal enhancement (only capsular and periurethral flow); score 2: probably benign, mild enhancement (symmetric radial flow from capsular branches); score 3: indeterminate, mildly increased enhancement (asymmetric/increased flow in the prostate); score 4: probably malignant, moderately increased enhancement (asymmetric/increased flow in the prostate); score 5: definitely malignant, substantially increased enhancement (asymmetric/increased flow in the prostate). We considered those TR-CDFI findings with mSBRS score ≥ 4 to be of PCA suspicion.

### Assessment of multi-parametric MRI

Patients of both centers received mpMRI scan with the same field strength (3.0T) and vendor (Siemens). The scan included the sequences of triplanar T2-weighted, dynamic contrast-enhanced, diffusion-weighted imaging, and MR spectroscopy, abiding existing protocols [8]. Despite initial scoring and analysis by multiple experienced practitioners, all mpMRI images were re-evaluated and scored by biopsy performer Dr. Lu and Dr. Liu (both with 10 years of experience in prostate MRI), in compliance with Prostate Imaging Reporting and Data System (PI-RADS) Version 2.1 [9]. MRI outcomes with PI-RADS score ≥ 3 were designated suspicious of PCA.

### Biopsy protocol and histopathological analysis

All biopsy procedures were performed utilizing a real-time dedicated ultrasound platform (Aloka pro-sound 3500). We integrated systematic biopsy (12 cores for each patient: apex, mid-gland and base, lateral and medial aspects of each sextant, on right and left lobes) and targeted biopsy (2–4 cores per target, up to 4 targets for each patient) for the process. Every systematic core and target set was collected in separated containers, for accurate localization of pathological findings. Biopsy samples were analyzed by two experienced pathologists from two centers, and reports included histological definitions according to the International Society of Urological Pathology (ISUP), Gleason score, percentage, and total length of core involvement by PCA for each core. We defined clinically insignificant PCA (cisPCA) as ISUP grade group 1 (Gleason score 3 + 3), disregarding numbers or percentage of cores involved. Conversely, clinically significant PCA (csPCA) was defined as tumors graded ISUP group 2 or higher (Gleason score ≥ 3 + 4). Highest ISUP (Gleason score) was used to grade the tumor should multiple cores presented with different gradings.

### PCA risk analysis and diagnostic pathways

**Fig. 1:**
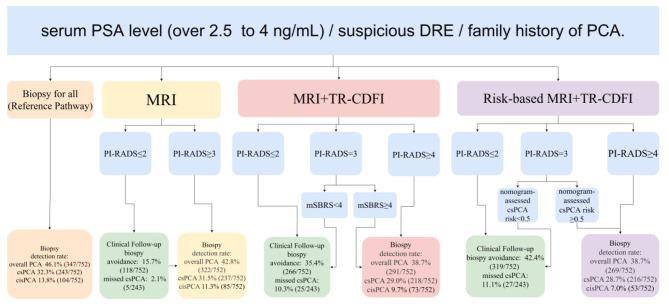
Flow chart of diagnostic pathways for men suspicious of PCA. Four diagnostic pathways (biopsy for all, MRI-directed, MRI-directed TR-CDFI and risk-based MRI-directed TR-CDFI pathways) are presented graphically, with respective outcomes listed in result boxes

Following the concept of risk-adapted MRI pathways put forth in recent literatures [10, 11], we constructed a dedicative nomogram via multivariate logistic regression analysis. A patient’s risk of PCA over 0.5 determined through nomogram was considered as “High risk”, as risk below 0.5 was classified into the “Low risk” subgroup. Afterwards, 4 diagnostic pathways were retrospectively designated and reviewed: (1) Biopsy all pathway (Reference pathway): Biopsy for all patients in the study; (2) MRI-directed pathway: Biopsy for patients with positive MRI findings (PI-RADS ≥ 3), and patients with negative MRI findings (PI-RADS < 3) would not undergo further diagnostic tests; (3) MRI-directed TR-CDFI pathway: Biopsy for patients with strong positive MRI findings (PI-RADS ≥ 4) or patients with positive MRI findings (PI-RADS = 3) and positive TR-CDFI findings (mSBRS ≥ 4), as those with negative MRI findings (PI-RADS < 3) or positive MRI findings (PI-RADS = 3) but negative TR-CDFI findings (mSBRS < 4) would not receive subsequent diagnostic tests. (4) Risk-based MRI-directed TR-CDFI pathway: Inspired the recently proposed risk-based prostate biopsy strategy [10], patients would be first assessed with the nomogram mentioned above and classified into high and low risk group. Then, based on the risk status, which already incorporated mSBRS scoring of TR-CDFI, combined with the patients’ MRI findings, candidates for biopsies would be selected. The details of all 4 diagnostic sequences were illustrated in Fig. 1.

### Outcome measurements and statistical analysis

Respective calculations on medians with interquartile ranges, frequencies, and proportions were made for continuous and categorical variables. We chose overall PCA detection rate, csPCA detection rate, cisPCA detection rate as well as biopsy avoidance rate and missed csPCA detection rate as the main outcomes of this study. Univariate logistic regression analysis was adopted to identify independent risk factor for the subsequent multivariate logistic regression analysis (enter mode was adopted), upon which a predictive nomogram would be produced. To better compare the performances of all diagnostic pathways, we utilized decision curve analysis [12], which focused on the net clinical benefits a patient could receive under different threshold probability levels, thus revealing the actual clinical impacts of certain procedures. Net benefit in our study could be calculated as “true positive rate – (false positive rate x weighting factor)”, where weighting factor equals “Threshold probability/1—threshold probability”. P-values < 0.05 were considered of statistical significance. All statistical analyses were conducted using R version 4.03, with rmda package.

## Results

### Overall outcomes

Median PSA level was 11.89 ng/mL, as median prostate volume was 80.92 mL and median PSAD was 0.15. It was shown that for the reference pathway (biopsy for all), PCA detection rate was 46.1% (347/752), while the detection rate of csPCA and cisPCA were 32.3% (243/752) and 13.8% (104/752) respectively. The distribution of mSBRS scoring were: mSBRS 1–3 (47.4%) and mSBRS 4–5 (52.6%), as the distribution of PI-RADS scores were: PI-RADS 1–2 (16.4%), PI-RADS 3 (40.9%) and PI-RADS 4–5 (42.7%). More detailed descriptive data were presented in Table 1.

### Outcomes of univariate, multivariate logistic regression analyses and the predictive nomogram

**Fig. 2:**
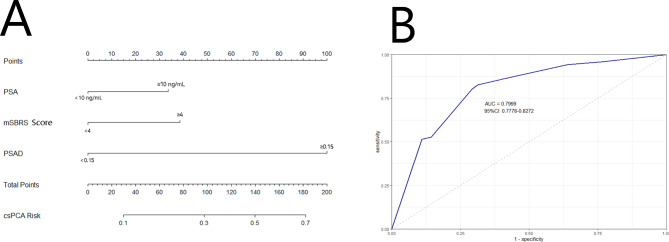
**(A)** predictive nomogram for clinically significant PCA (csPCA). **(B)** Receiver-operating curve of the predictive nomogram

Risk factors of Prostate Specific Antigen (PSA) > 10 ng/mL, Prostate Specific Antigen density (PSAD) > 0.15 and mSBRS score ≥ 4 were identified as potentially risk factors of csPCA in univariate logistic regression analysis (all with p-value < 0.001). Further multivariate logistic regression showed all three risk factors to be independent risk factors of csPCA (PSAD > 0.15 and mSBRS score ≥ 4: p < 0.001; PSA > 10: p = 0.0128). A predictive nomogram was thus built utilizing these results, as demonstrated in Fig. 2A. Subsequent ROC analysis based internal validation presented an acceptable result (area under curve = 0.7999, as depicted in Fig. 2B). Using this predictive tool, we were able to separate all cases into high (PCA risk ≥ 0.5) and low (PCA risk<0.5) risk group, as presented in Table 1.

### Outcomes of diagnostic pathways

Graphical representations of the outcomes were illustrated in Fig. 1. The reference pathway’s outcomes had been presented in the above section, with biopsy avoidance and missed csPCA detection rate being 0. For the MRI-directed pathway: overall PCA detection rate was 42.8% (322/752), while the csPCA and cisPCA detection rates were 31.5% (237/752) and 11.3% (85/752). Biopsy avoidance rate was 15.7% (118/752), as missed csPCA detection rate was 2.1% (5/243). For the MRI-directed TR-CDFI pathway: combined PCA detection rate was 38.7% (291/752), the csPCA detection rate was 29.0% (218/752) and the cisPCA detection rate was 9.7% (73/752). Meanwhile, Biopsy avoidance rate was 35.4% (266/752), and missed csPCA detection rate was 10.3% (25/243). For the Risk-based MRI-directed TR-CDFI pathway: PCA detection rate was 38.7% (269/752), as the csPCA detection rate was 28.7% (216/752) and the cisPCA detection rate was 7.0% (53/752). Biopsy avoidance rate was 42.4% (319/752), and 27 out of 243 (11.1%) of csPCA patients would be missed.

### Outcomes of decision curve analysis

**Fig. 3:**
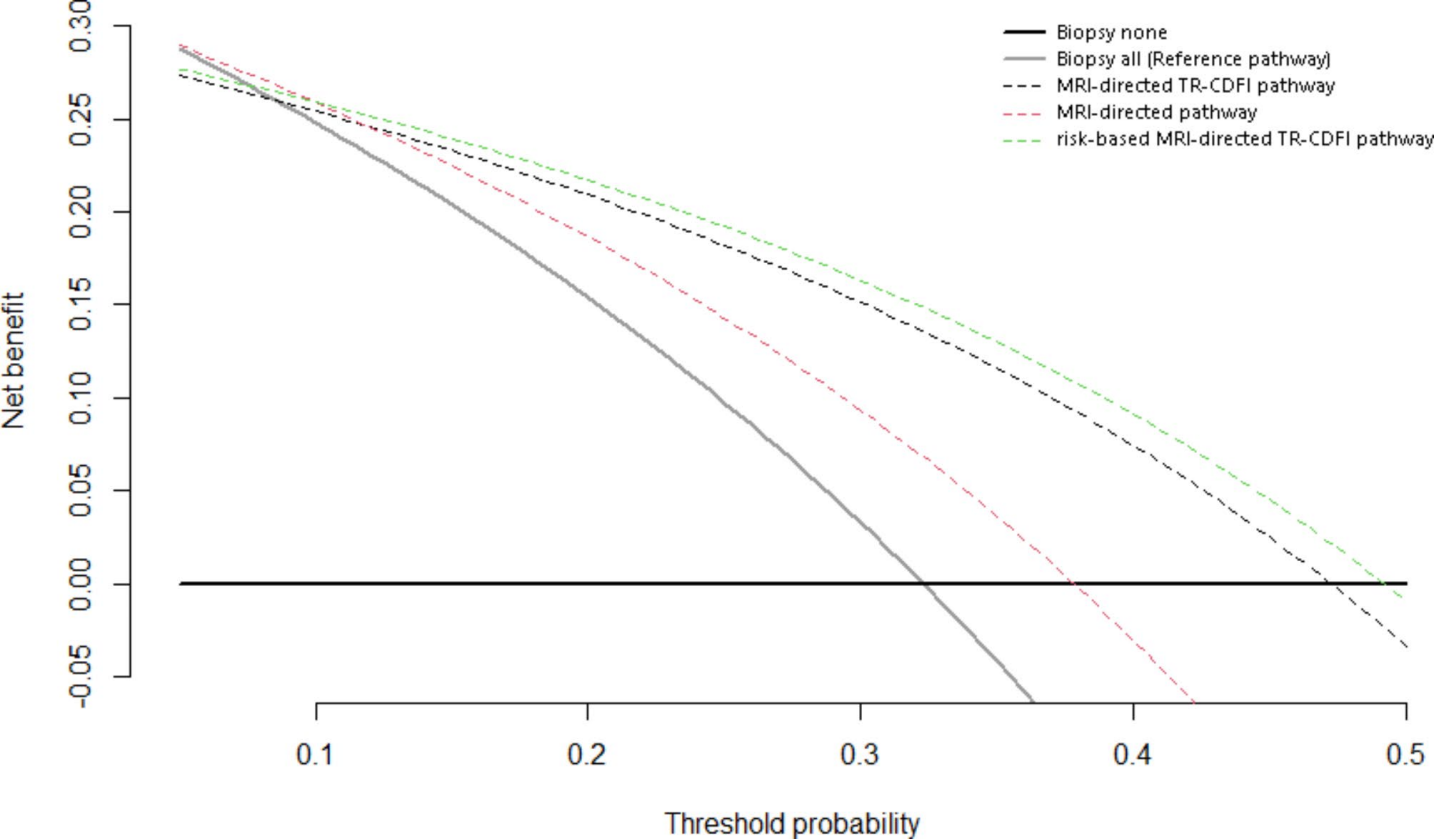
Decision curve analysis comparing the net benefits of different diagnostic pathways showed that the risk-based MRI directed TR-CDFI pathway had the highest net benefit between the threshold probability level of 0.1 and 0.5

Decision curve analysis (depicted in Fig. 3) revealed that the MRI-directed pathway produced the highest net benefit between the threshold probability level 0 to 0.10, while between 0.10 and 0.51, risk-based MRI-directed TR-CDFI pathway showed superiority over all other strategies. It was also noteworthy that MRI-directed TR-CDFI pathway surpassed the MRI-directed pathway at the threshold probability level of 0.12 in term of net benefit.

## Discussion

TR-CDFI as a more familiar diagnostic technique compared to mpMRI has demonstrated superior traits in terms of patient’s medical expenditures and waiting time [13]. Moreover, its availability among the vast majority of rudimentary medical centers in China further increased its accessibility to patients, thus boosting their willingness to receive this kind of diagnostic test. Considering that PCA patients in China are less likely to take part in early screenings and thus more likely to be diagnosed with later stages of the disease [14], introducing accessible and non-invasive diagnostic tools (including serum PSA test and TR-CDFI) that promote earlier diagnosis could likely bring more benefits, as demonstrated in an recent Italian literature [15]. Combing with the fact that mpMRI’s high sensitivity were likely to present false positive results leading to unnecessary biopsies, we were convinced that incorporating TR-CDFI into the current PCA biopsy strategies which are predominately mpMRI-centered could potentially enhance their performances.

The overall results of our study in terms of PCA (46.1%), csPCA (32.3%) and cisPCA (13.8%) detection rates are generally in resemblances with recent Randomized clinical trials and meta-analyses (Drost et al. reported in a 5000 patients meta-analysis where overall PCA detection rate was 49%, as csPCA and cisPCA detection rates were 28% and 21% respectively) [16, 17]. This suggested that the results of our study could potentially serve as references when validating the prostate biopsy outcomes of other medical centers. Furthermore, due to the fact that all of the patients enrolled received surgical intervention, and had their removed prostate tissue histo-pathologically examined, the grading of PCA in our study bore more accuracy compared to the researches where grading was according to biopsy alone [7, 18]. In comparison with existing studies’ risk stratification methods [7, 18], we utilized a predictive nomogram that quantified the risk of csPCA with data from the cohort itself, which in our belief would better characterize the likelihood of csPCA in each individual patient. PSA, PSAD and mSBRS score were incorporated into the predictive model as a result of the univariate and multivariate regression analyses. The cut-off value of PSA level of 10 ng/mL was chosen based on the risk stratification of D’Amico et al.’s research [19], where patients with PSA > 10 ng/mL were sub-grouped into intermediate or high biochemical recurrence risk groups. PSAD over 0.15 on the other hand had been adopted in known literatures [20, 21] as a predictor of csPCA. Though without wide applications, mSBRS score had shown acceptable preliminary outcomes in predicting the pathological grade of PCA biopsy [6] as well as the biochemical recurrence [22]. These combined justified the use of our predictive model as a preliminary risk-stratification tool for csPCA.

Decision curve analysis had been applied and recommended in a number of prostate-biopsy- related thesis [23–25]. Threshold probability under the scenario of prostate biopsy could be expressed as the physician’s willingness to carry out a certain number of biopsies in order to find csPCA, as exemplified in the work of Vickers et.al [23]. If a urologist agrees with conducting 10 biopsies, then the threshold probability would be 0.1. It could also be sparsely concluded that if one diagnostic pathway holds the highest net benefit under a given range of threshold probability, that particular strategy would most likely bring the most clinical benefits to the patients. In our study, we noticed that between the threshold probability level of 0.1 and 0.5, the risk-based MRI-directed TR-CDFI pathway held the highest net benefit. It should be reminded that for each suspicious PCA target, our combined SBx and TBx approach took from 2 to 12 samples, suggesting that for most patients in this cohort, the realistic range of threshold probability would be between 0.1 and 0.5. Consequently, it could be concluded that in our research the risk-based MRI-directed TR-CDFI pathway that combined TR-CDFI and risk-stratification nomogram out-performed other pathways in terms of clinical benefits.

Although our study has achieved progress in assessing the possible role TR-CDFI and risk-stratification nomogram could play in a MRI-directed biopsy pathway, it simultaneously bore flaws that could potentially hinder our efforts. Firstly, our research was retrospective in nature, thus requiring additional retrospective and prospective studies to assess the consistency of observations and application in actual clinical decision-making, over a range of disease prevalence. Secondarily, all of the patients in our thesis opted for surgical intervention. Though the prevalence rate of csPCA was not substantially different from previous researches’ findings, it could be deduced that patients in this cohort might also be different from the broader population of men, perhaps with severer symptoms, suggesting further studies on a larger population. Thirdly, histo-pathological analysis based on the tissue obtained from enucleation of prostate might not accurately reflect the true pathological grading of the patient’s prostate, thus lowering the overall creditability of our works.

## Conclusions

To summarize, the present study demonstrated that MRI-directed diagnostic pathway combined with risk-stratification and TR-CDFI out-performed other strategies under a realistic range of threshold probability, balancing csPCa detection with biopsy and cisPCa overdiagnosis avoidance, thus reducing the chances of unnecessary biopsies. This would further reinforce the concept of “risk-based” MRI-directed biopsy decisions, and simultaneously propose TR-CDFI’s new role in the screening and early diagnosis of csPCA.

## Electronic supplementary material

Below is the link to the electronic supplementary material.

Additional File: Supplementary Table 1 baseline characteristics of the patients ?with patient ID omitted)

## Data Availability

All data generated or analysed during this study are included in this published article (supplementary material 1, all patients’ names were omitted).

## List of Abbreviations

cisPCA: Clinically-insignificant Prostate Cancer
csPCA: Clinically-significant Prostate Cancer
IQR: Interquartile range
Mp-MRI: Multi-parametric Magnetic Resonance Imaging
mSBRS: Modified Subjective Blood-flow Rating Scale.
PCA: Prostate Cancer
PSA: Prostate-specific Antigen
PSAD: Prostate-specific Antigen Density
ROC: Receiver-Operating cure
SBx: Systemic Biopsy
TBx: Targeted Biopsy
TR-CDFI: Trans-rectal Color Doppler Flow Imaging
