# Supplementary material for: Incorporation of trans-rectal color doppler flow imaging and risk-stratification nomogram reduce unnecessary prostate biopsies in suspected prostate cancer patients: a bi-centered retrospective validation study
Source: BMC Urol. 2023 May 3;23:81. doi: 10.1186/s12894-023-01245-2 (PMC10157911; doi:10.1186/s12894-023-01245-2)
Supplement: Supplementary file 1 — Additional File: Supplementary Table 1 baseline characteristics of the patients ?with patient ID omitted) [file 12894_2023_1245_MOESM1_ESM.docx]

## Supplementary Table 1. baseline characteristics of the patients （with patient ID omitted）

**csPCA: clinical significant prostate cancer (Gleason Score ≥ 4+3，), with 1 indicating having diagnosed with csPCA**

**PSA: Prostate Specific Antigen**

**PSV Prostate volume**

**PIRADS: Prostate Imaging Reporting and Data System score**

**mSBRS: modified Subjective Blood-flow Rating Scale, with 1 indicating having mSBRS over 4**

**PSAD: Prostate-specific Antigen Density**

**Combined PCA: both clinical significant and insignificant prostate cancer, with 1 indicating having diagnosed with either csPCA or cisPCA**

| csPCA | PSA | PSV | PSAD | PIRADS | mSBRS≥4 | Combined PCA |
| --- | --- | --- | --- | --- | --- | --- |
| 0 | 3.851 | 49 | 0.078591837 | 3 | 1 | 1 |
| 1 | 16.845 | 48 | 0.3509375 | 5 | 0 | 1 |
| 0 | 10.675 | 49 | 0.217857143 | 3 | 0 | 0 |
| 1 | 45.504 | 62 | 0.733935484 | 3 | 0 | 1 |
| 0 | 49.867 | 75 | 0.664893333 | 3 | 0 | 0 |
| 0 | 0.929 | 27 | 0.034407407 | 4 | 0 | 0 |
| 1 | 51.996 | 66 | 0.787818182 | 4 | 0 | 1 |
| 1 | 43.845 | 43 | 1.019651163 | 5 | 1 | 1 |
| 0 | 48.151 | 88 | 0.547170455 | 3 | 0 | 0 |
| 0 | 10.209 | 107 | 0.095411215 | 2 | 0 | 0 |
| 0 | 9.047 | 50 | 0.18094 | 4 | 0 | 0 |
| 0 | 5.315 | 40 | 0.132875 | 2 | 0 | 1 |
| 0 | 16.095 | 90 | 0.178833333 | 4 | 0 | 0 |
| 0 | 14.04 | 109 | 0.128807339 | 3 | 0 | 0 |
| 0 | 6.073 | 70 | 0.086757143 | 3 | 0 | 0 |
| 0 | 8.967 | 79 | 0.113506329 | 2 | 0 | 0 |
| 0 | 15.274 | 87 | 0.175563218 | 2 | 0 | 0 |
| 1 | 10.218 | 40 | 0.25545 | 4 | 1 | 1 |
| 0 | 25.996 | 93 | 0.279526882 | 3 | 1 | 0 |
| 0 | 21.726 | 129 | 0.168418605 | 4 | 1 | 0 |
| 0 | 13.276 | 78 | 0.170205128 | 5 | 1 | 0 |
| 0 | 28.261 | 142 | 0.199021127 | 3 | 1 | 1 |
| 0 | 6.097 | 78 | 0.078166667 | 3 | 1 | 0 |
| 0 | 6.273 | 73 | 0.085931507 | 2 | 1 | 0 |
| 0 | 6.735 | 85 | 0.079235294 | 3 | 1 | 0 |
| 1 | 7.553 | 59 | 0.128016949 | 4 | 1 | 1 |
| 0 | 8.932 | 76 | 0.117526316 | 2 | 0 | 0 |
| 1 | 12.769 | 46 | 0.277586957 | 5 | 1 | 1 |
| 0 | 2.313 | 35 | 0.066085714 | 4 | 1 | 1 |
| 0 | 14.602 | 75 | 0.194693333 | 3 | 0 | 0 |
| 1 | 1288.843 | 72 | 17.90059722 | 5 | 1 | 1 |
| 0 | 31.58 | 77 | 0.41012987 | 3 | 0 | 0 |
| 1 | 15.406 | 25 | 0.61624 | 4 | 0 | 1 |
| 0 | 4.064 | 55 | 0.073890909 | 3 | 1 | 0 |
| 0 | 5.226 | 77 | 0.06787013 | 3 | 1 | 1 |
| 1 | 21.609 | 34 | 0.635558824 | 3 | 1 | 1 |
| 0 | 4.961 | 105 | 0.047247619 | 3 | 1 | 0 |
| 0 | 8.811 | 67 | 0.131507463 | 2 | 0 | 0 |
| 1 | 12 | 99 | 0.121212121 | 4 | 1 | 1 |
| 1 | 71.052 | 53 | 1.340603774 | 4 | 0 | 1 |
| 0 | 7.255 | 103 | 0.070436893 | 4 | 1 | 1 |
| 0 | 8.456 | 124 | 0.068193548 | 3 | 0 | 0 |
| 0 | 33.858 | 92 | 0.368021739 | 3 | 1 | 0 |
| 0 | 7.489 | 68 | 0.110132353 | 3 | 1 | 1 |
| 1 | 9 | 23 | 0.391304348 | 4 | 1 | 1 |
| 0 | 12.74 | 105 | 0.121333333 | 3 | 0 | 0 |
| 0 | 17.728 | 65 | 0.272738462 | 4 | 0 | 1 |
| 0 | 5.602 | 37 | 0.151405405 | 3 | 1 | 0 |
| 1 | 18 | 29 | 0.620689655 | 4 | 1 | 1 |
| 0 | 14.086 | 38 | 0.370684211 | 5 | 1 | 1 |
| 0 | 7.059 | 87 | 0.081137931 | 3 | 1 | 1 |
| 1 | 15.76 | 82 | 0.192195122 | 4 | 1 | 1 |
| 0 | 14.12 | 110 | 0.128363636 | 3 | 1 | 1 |
| 0 | 1.978 | 58 | 0.034103448 | 3 | 1 | 0 |
| 0 | 12.314 | 96 | 0.128270833 | 4 | 1 | 1 |
| 0 | 18.54 | 37 | 0.501081081 | 3 | 1 | 0 |
| 0 | 9.048 | 46 | 0.196695652 | 2 | 1 | 0 |
| 0 | 1.532 | 61 | 0.025114754 | 2 | 0 | 0 |
| 0 | 29.681 | 220 | 0.134913636 | 3 | 1 | 1 |
| 0 | 8.811 | 67 | 0.131507463 | 5 | 0 | 1 |
| 1 | 299.17 | 119 | 2.514033613 | 5 | 1 | 1 |
| 0 | 5.37 | 59 | 0.091016949 | 2 | 1 | 0 |
| 0 | 10.779 | 111 | 0.097108108 | 3 | 1 | 0 |
| 0 | 6.857 | 49 | 0.139938776 | 2 | 0 | 0 |
| 0 | 9.291 | 101 | 0.091990099 | 3 | 0 | 1 |
| 1 | 9.27 | 64 | 0.14484375 | 5 | 0 | 1 |
| 0 | 8.862 | 68 | 0.130323529 | 3 | 1 | 0 |
| 0 | 7.266 | 105 | 0.0692 | 3 | 0 | 0 |
| 0 | 3.764 | 86 | 0.043767442 | 2 | 1 | 0 |
| 0 | 7.969 | 118 | 0.067533898 | 3 | 1 | 0 |
| 0 | 12.831 | 67 | 0.191507463 | 2 | 0 | 0 |
| 0 | 9.342 | 107 | 0.087308411 | 3 | 1 | 0 |
| 0 | 11.422 | 81 | 0.141012346 | 3 | 0 | 0 |
| 0 | 13.104 | 65 | 0.2016 | 2 | 1 | 0 |
| 1 | 50.661 | 68 | 0.745014706 | 5 | 0 | 1 |
| 0 | 1.14 | 21 | 0.054285714 | 3 | 1 | 1 |
| 0 | 3.298 | 60 | 0.054966667 | 3 | 1 | 0 |
| 0 | 14.437 | 54 | 0.267351852 | 3 | 1 | 1 |
| 0 | 0.759 | 90 | 0.008433333 | 1 | 1 | 0 |
| 0 | 9.42 | 90 | 0.104666667 | 2 | 0 | 0 |
| 0 | 11.602 | 90 | 0.128911111 | 2 | 0 | 0 |
| 1 | 6.684 | 27 | 0.247555556 | 4 | 0 | 1 |
| 0 | 7.173 | 50 | 0.14346 | 4 | 1 | 0 |
| 0 | 15.446 | 95 | 0.162589474 | 3 | 0 | 0 |
| 0 | 1.059 | 47 | 0.022531915 | 2 | 1 | 0 |
| 0 | 10.995 | 70 | 0.157071429 | 4 | 0 | 0 |
| 0 | 8.393 | 30 | 0.279766667 | 3 | 1 | 0 |
| 0 | 6 | 59 | 0.101694915 | 3 | 1 | 0 |
| 0 | 7.016 | 112 | 0.062642857 | 3 | 1 | 0 |
| 0 | 7.504 | 112 | 0.067 | 4 | 0 | 0 |
| 1 | 3.323 | 43 | 0.07727907 | 3 | 1 | 1 |
| 0 | 0.641 | 30 | 0.021366667 | 2 | 0 | 0 |
| 0 | 8.891 | 45 | 0.197577778 | 3 | 1 | 0 |
| 0 | 7.775 | 88 | 0.088352273 | 4 | 1 | 1 |
| 1 | 188.011 | 93 | 2.021623656 | 5 | 1 | 1 |
| 1 | 17.546 | 15 | 1.169733333 | 4 | 1 | 1 |
| 0 | 3.061 | 60 | 0.051016667 | 2 | 0 | 1 |
| 0 | 12.127 | 39 | 0.310948718 | 4 | 0 | 0 |
| 0 | 3.846 | 77 | 0.049948052 | 2 | 1 | 0 |
| 0 | 10.636 | 85 | 0.125129412 | 4 | 0 | 1 |
| 1 | 10.523 | 21 | 0.501095238 | 4 | 0 | 1 |
| 1 | 98.653 | 60 | 1.644216667 | 5 | 0 | 1 |
| 0 | 81.987 | 82 | 0.999841463 | 2 | 0 | 0 |
| 0 | 4.489 | 58 | 0.077396552 | 3 | 1 | 0 |
| 1 | 5.104 | 30 | 0.170133333 | 3 | 1 | 1 |
| 0 | 9.551 | 60 | 0.159183333 | 4 | 1 | 0 |
| 0 | 16.297 | 64 | 0.254640625 | 2 | 1 | 0 |
| 1 | 100 | 115 | 0.869565217 | 4 | 1 | 1 |
| 1 | 13.478 | 132 | 0.102106061 | 4 | 1 | 1 |
| 0 | 7.656 | 63 | 0.12152381 | 3 | 1 | 0 |
| 1 | 25.757 | 30 | 0.858566667 | 4 | 1 | 1 |
| 1 | 11.506 | 108 | 0.106537037 | 5 | 1 | 1 |
| 0 | 0.768 | 35 | 0.021942857 | 4 | 0 | 0 |
| 0 | 4.407 | 82 | 0.053743902 | 2 | 1 | 0 |
| 1 | 1.693 | 25 | 0.06772 | 4 | 1 | 1 |
| 1 | 32.815 | 40 | 0.820375 | 4 | 1 | 1 |
| 1 | 98.197 | 73 | 1.345164384 | 4 | 1 | 1 |
| 1 | 89.621 | 42 | 2.133833333 | 5 | 1 | 1 |
| 0 | 11.508 | 104 | 0.110653846 | 4 | 1 | 0 |
| 0 | 41.258 | 68 | 0.606735294 | 3 | 0 | 0 |
| 0 | 5.867 | 43 | 0.13644186 | 5 | 1 | 1 |
| 1 | 31.911 | 24 | 1.329625 | 4 | 1 | 1 |
| 0 | 10.228 | 92 | 0.111173913 | 4 | 1 | 1 |
| 0 | 4.13 | 35 | 0.118 | 3 | 0 | 0 |
| 1 | 0.977 | 30 | 0.032566667 | 4 | 0 | 1 |
| 1 | 56.742 | 48 | 1.182125 | 4 | 1 | 1 |
| 0 | 16.466 | 84 | 0.19602381 | 2 | 1 | 0 |
| 0 | 12.192 | 43 | 0.283534884 | 1 | 1 | 0 |
| 1 | 9.432 | 81 | 0.116444444 | 3 | 0 | 0 |
| 0 | 5.802 | 111 | 0.05227027 | 4 | 0 | 0 |
| 1 | 28.408 | 33 | 0.860848485 | 5 | 1 | 1 |
| 0 | 6.753 | 50 | 0.13506 | 4 | 1 | 0 |
| 0 | 12.317 | 87 | 0.141574713 | 2 | 1 | 0 |
| 0 | 17.816 | 120 | 0.148466667 | 4 | 1 | 0 |
| 0 | 4.885 | 113 | 0.043230088 | 3 | 0 | 0 |
| 0 | 5.937 | 51 | 0.116411765 | 3 | 1 | 1 |
| 0 | 11.176 | 58 | 0.192689655 | 2 | 0 | 0 |
| 0 | 16.279 | 112 | 0.145348214 | 2 | 1 | 1 |
| 0 | 10.323 | 76 | 0.135828947 | 3 | 0 | 1 |
| 0 | 63.117 | 121 | 0.521628099 | 2 | 1 | 0 |
| 0 | 13.402 | 33 | 0.406121212 | 4 | 1 | 1 |
| 0 | 12.733 | 54 | 0.235796296 | 2 | 0 | 0 |
| 0 | 4.727 | 98 | 0.048234694 | 3 | 1 | 0 |
| 0 | 14.755 | 54 | 0.273240741 | 4 | 1 | 0 |
| 0 | 6.261 | 55 | 0.113836364 | 4 | 1 | 0 |
| 0 | 7.32 | 26 | 0.281538462 | 2 | 0 | 0 |
| 1 | 81.367 | 21 | 3.874619048 | 4 | 1 | 1 |
| 0 | 48.29 | 60 | 0.804833333 | 4 | 0 | 1 |
| 0 | 41.102 | 31 | 1.325870968 | 3 | 0 | 0 |
| 0 | 0.803 | 16 | 0.0501875 | 2 | 1 | 0 |
| 1 | 89.082 | 13.5 | 6.598666667 | 3 | 1 | 1 |
| 0 | 8.576 | 73 | 0.117479452 | 1 | 0 | 0 |
| 0 | 6.706 | 71 | 0.094450704 | 3 | 0 | 0 |
| 0 | 5.873 | 67 | 0.087656716 | 3 | 1 | 0 |
| 0 | 7.419 | 53 | 0.139981132 | 2 | 1 | 0 |
| 0 | 13.604 | 108 | 0.125962963 | 2 | 1 | 0 |
| 1 | 10.164 | 51 | 0.199294118 | 4 | 0 | 1 |
| 0 | 3.839 | 48 | 0.079979167 | 2 | 1 | 0 |
| 0 | 6.53 | 31 | 0.210645161 | 3 | 1 | 1 |
| 0 | 7.77 | 32 | 0.2428125 | 2 | 1 | 0 |
| 0 | 10.574 | 49 | 0.215795918 | 4 | 0 | 0 |
| 0 | 0.549 | 28 | 0.019607143 | 4 | 0 | 0 |
| 0 | 12.513 | 58 | 0.215741379 | 4 | 0 | 0 |
| 0 | 27.767 | 55 | 0.504854545 | 2 | 0 | 0 |
| 0 | 6.073 | 70 | 0.086757143 | 1 | 1 | 0 |
| 1 | 16.889 | 29 | 0.58237931 | 4 | 1 | 1 |
| 0 | 11.188 | 57 | 0.196280702 | 4 | 1 | 1 |
| 0 | 8.308 | 21 | 0.395619048 | 4 | 0 | 0 |
| 0 | 15.012 | 92 | 0.163173913 | 4 | 0 | 0 |
| 1 | 9.006 | 30 | 0.3002 | 2 | 0 | 1 |
| 0 | 26.956 | 38 | 0.709368421 | 4 | 1 | 0 |
| 1 | 26.064 | 58 | 0.44937931 | 5 | 1 | 1 |
| 0 | 5.273 | 28 | 0.188321429 | 4 | 0 | 1 |
| 0 | 9.665 | 44 | 0.219659091 | 2 | 1 | 0 |
| 0 | 5.25 | 21 | 0.25 | 3 | 1 | 1 |
| 1 | 9.255 | 48 | 0.1928125 | 5 | 0 | 1 |
| 0 | 10.443 | 35 | 0.298371429 | 3 | 0 | 0 |
| 0 | 7.232 | 34 | 0.212705882 | 2 | 0 | 0 |
| 0 | 7.129 | 64 | 0.111390625 | 3 | 0 | 0 |
| 0 | 32.291 | 55 | 0.587109091 | 2 | 0 | 0 |
| 1 | 70.36 | 42 | 1.675238095 | 3 | 0 | 1 |
| 0 | 8.432 | 93 | 0.090666667 | 5 | 0 | 0 |
| 0 | 15.019 | 63 | 0.238396825 | 3 | 0 | 0 |
| 0 | 6.161 | 76 | 0.081065789 | 2 | 0 | 0 |
| 0 | 9.563 | 43 | 0.222395349 | 2 | 0 | 0 |
| 0 | 8.912 | 55 | 0.162036364 | 2 | 0 | 0 |
| 1 | 14.624 | 47 | 0.311148936 | 4 | 0 | 1 |
| 0 | 4.565 | 79 | 0.05778481 | 3 | 0 | 0 |
| 0 | 8.951 | 89 | 0.100573034 | 4 | 0 | 0 |
| 0 | 2.907 | 32 | 0.09084375 | 4 | 0 | 0 |
| 0 | 8.352 | 93 | 0.089806452 | 4 | 0 | 0 |
| 0 | 12.452 | 70 | 0.177885714 | 3 | 0 | 0 |
| 0 | 6.607 | 68 | 0.097161765 | 3 | 0 | 1 |
| 0 | 5 | 30 | 0.166666667 | 3 | 0 | 0 |
| 0 | 19.757 | 63 | 0.313603175 | 4 | 0 | 1 |
| 0 | 6.286 | 59 | 0.106542373 | 4 | 0 | 0 |
| 0 | 12.293 | 77 | 0.159649351 | 3 | 0 | 0 |
| 0 | 8.05 | 45 | 0.178888889 | 2 | 0 | 0 |
| 0 | 14.361 | 58 | 0.247603448 | 3 | 0 | 0 |
| 0 | 13.735 | 38 | 0.361447368 | 4 | 0 | 0 |
| 1 | 4.736 | 32 | 0.148 | 3 | 0 | 1 |
| 1 | 35.342 | 21 | 1.682952381 | 4 | 0 | 1 |
| 0 | 11.814 | 125 | 0.094512 | 4 | 0 | 1 |
| 0 | 16.269 | 153 | 0.106333333 | 3 | 0 | 1 |
| 1 | 33.433 | 25 | 1.33732 | 3 | 0 | 1 |
| 0 | 4.499 | 100 | 0.04499 | 3 | 0 | 0 |
| 0 | 2.702 | 74 | 0.036513514 | 2 | 0 | 0 |
| 0 | 11.661 | 59 | 0.197644068 | 2 | 0 | 0 |
| 1 | 20.327 | 27 | 0.752851852 | 5 | 0 | 1 |
| 1 | 12.102 | 13 | 0.930923077 | 4 | 0 | 1 |
| 0 | 28.981 | 158 | 0.183424051 | 2 | 0 | 1 |
| 0 | 5.925 | 80 | 0.0740625 | 2 | 0 | 0 |
| 0 | 19.501 | 65 | 0.300015385 | 2 | 0 | 0 |
| 1 | 70.478 | 30 | 2.349266667 | 5 | 0 | 1 |
| 0 | 46.921 | 65 | 0.721861538 | 3 | 0 | 0 |
| 0 | 16.652 | 80 | 0.20815 | 3 | 0 | 0 |
| 0 | 22.04 | 28 | 0.787142857 | 3 | 0 | 0 |
| 0 | 9.876 | 69 | 0.143130435 | 3 | 0 | 1 |
| 0 | 12.612 | 61 | 0.206754098 | 3 | 0 | 1 |
| 0 | 10.322 | 81 | 0.127432099 | 3 | 0 | 0 |
| 0 | 5.839 | 61 | 0.095721311 | 3 | 0 | 0 |
| 0 | 2.308 | 57 | 0.040491228 | 4 | 0 | 0 |
| 1 | 940.92 | 90 | 10.45466667 | 4 | 0 | 1 |
| 0 | 5.992 | 56 | 0.107 | 2 | 1 | 1 |
| 1 | 74.533 | 59 | 1.263271186 | 4 | 1 | 1 |
| 0 | 9.925 | 89 | 0.111516854 | 3 | 1 | 0 |
| 0 | 1.656 | 32 | 0.05175 | 3 | 1 | 0 |
| 0 | 7.122 | 49 | 0.145346939 | 2 | 1 | 1 |
| 1 | 18.702 | 25 | 0.74808 | 3 | 1 | 1 |
| 0 | 4.971 | 50 | 0.09942 | 5 | 1 | 1 |
| 0 | 5.277 | 74 | 0.071310811 | 3 | 1 | 0 |
| 0 | 5.973 | 110 | 0.0543 | 2 | 1 | 1 |
| 0 | 9.281 | 53 | 0.175113208 | 4 | 1 | 0 |
| 0 | 12.28 | 122 | 0.100655738 | 2 | 1 | 1 |
| 0 | 8.207 | 123 | 0.066723577 | 3 | 1 | 1 |
| 1 | 10.78 | 43 | 0.250697674 | 4 | 1 | 1 |
| 0 | 4.306 | 75 | 0.057413333 | 3 | 1 | 1 |
| 0 | 4.28 | 31 | 0.138064516 | 3 | 1 | 0 |
| 0 | 6.875 | 43 | 0.159883721 | 2 | 1 | 1 |
| 0 | 46.867 | 36 | 1.301861111 | 2 | 1 | 1 |
| 0 | 11.489 | 57 | 0.201561404 | 2 | 1 | 1 |
| 1 | 44.051 | 38 | 1.159236842 | 5 | 1 | 1 |
| 0 | 9.029 | 66 | 0.13680303 | 1 | 1 | 1 |
| 0 | 2.138 | 58 | 0.036862069 | 3 | 1 | 0 |
| 0 | 1.586 | 31 | 0.05116129 | 3 | 1 | 0 |
| 0 | 8.625 | 140 | 0.061607143 | 4 | 1 | 0 |
| 1 | 19.617 | 33 | 0.594454545 | 4 | 1 | 1 |
| 0 | 23.734 | 109 | 0.217743119 | 4 | 1 | 1 |
| 0 | 25.94 | 45 | 0.576444444 | 2 | 1 | 1 |
| 1 | 17.835 | 58 | 0.3075 | 5 | 1 | 1 |
| 0 | 6.526 | 72 | 0.090638889 | 2 | 1 | 0 |
| 1 | 18.07 | 30 | 0.602333333 | 4 | 1 | 1 |
| 0 | 7.03 | 48 | 0.146458333 | 2 | 1 | 1 |
| 1 | 25.198 | 61 | 0.413081967 | 4 | 1 | 1 |
| 0 | 6.111 | 27 | 0.226333333 | 3 | 1 | 1 |
| 1 | 430.248 | 18 | 23.90266667 | 4 | 1 | 1 |
| 0 | 4.055 | 52 | 0.077980769 | 4 | 1 | 1 |
| 1 | 29.939 | 37 | 0.809162162 | 4 | 1 | 1 |
| 0 | 10.415 | 98 | 0.10627551 | 4 | 1 | 1 |
| 0 | 11.488 | 67 | 0.171462687 | 4 | 1 | 1 |
| 0 | 4.528 | 51 | 0.088784314 | 3 | 1 | 1 |
| 1 | 25.705 | 72 | 0.357013889 | 4 | 1 | 1 |
| 0 | 8.631 | 40 | 0.215775 | 3 | 1 | 0 |
| 1 | 16.653 | 35 | 0.4758 | 4 | 1 | 1 |
| 0 | 14.754 | 108 | 0.136611111 | 4 | 1 | 0 |
| 0 | 8.633 | 52 | 0.166019231 | 3 | 1 | 1 |
| 0 | 3.428 | 67 | 0.051164179 | 3 | 1 | 1 |
| 0 | 22.012 | 59 | 0.373084746 | 4 | 1 | 1 |
| 0 | 15.415 | 129 | 0.119496124 | 3 | 1 | 0 |
| 1 | 3.627 | 71 | 0.051084507 | 5 | 1 | 1 |
| 0 | 85.506 | 266 | 0.321451128 | 5 | 1 | 1 |
| 0 | 11.381 | 117 | 0.097273504 | 4 | 0 | 0 |
| 1 | 11.982 | 15 | 0.7988 | 4 | 1 | 1 |
| 0 | 12.124 | 56 | 0.2165 | 2 | 0 | 1 |
| 1 | 91 | 189 | 0.481481481 | 5 | 1 | 1 |
| 0 | 9.583 | 19.2 | 0.499114583 | 2 | 1 | 0 |
| 1 | 250 | 84.5 | 2.958579882 | 5 | 1 | 1 |
| 0 | 1.406 | 89.8 | 0.015657016 | 3 | 0 | 0 |
| 1 | 7.182 | 84.3 | 0.08519573 | 4 | 1 | 1 |
| 1 | 140 | 93 | 1.505376344 | 4 | 1 | 1 |
| 1 | 275 | 100 | 2.75 | 4 | 0 | 1 |
| 0 | 41.75 | 81 | 0.515432099 | 4 | 1 | 0 |
| 0 | 11.386 | 60.4 | 0.188509934 | 4 | 0 | 0 |
| 1 | 8.852 | 116.9 | 0.07572284 | 4 | 1 | 1 |
| 0 | 9.451 | 95.7 | 0.098756531 | 3 | 0 | 0 |
| 1 | 17.54 | 90.6 | 0.193598234 | 3 | 1 | 1 |
| 0 | 7.491 | 274.2 | 0.027319475 | 2 | 0 | 0 |
| 1 | 21.37 | 120 | 0.178083333 | 4 | 0 | 1 |
| 1 | 16.322 | 89.2 | 0.182982063 | 4 | 1 | 1 |
| 0 | 8.709 | 89.3 | 0.097525196 | 3 | 1 | 0 |
| 0 | 9.812 | 327.7 | 0.02994202 | 3 | 0 | 0 |
| 1 | 167.961 | 126 | 1.33302381 | 4 | 1 | 1 |
| 0 | 14.245 | 58.6 | 0.243088737 | 2 | 1 | 0 |
| 0 | 7.56 | 136.3 | 0.055465884 | 2 | 1 | 0 |
| 0 | 7.736 | 90.6 | 0.085386313 | 3 | 0 | 0 |
| 0 | 1.702 | 67.7 | 0.025140325 | 3 | 0 | 0 |
| 1 | 19.65 | 51.2 | 0.383789063 | 4 | 1 | 1 |
| 0 | 37.731 | 233.3 | 0.16172739 | 3 | 0 | 0 |
| 0 | 6 | 52.4 | 0.114503817 | 4 | 1 | 0 |
| 1 | 6.844 | 47 | 0.145617021 | 5 | 0 | 1 |
| 0 | 11.906 | 189.7 | 0.062762256 | 3 | 0 | 0 |
| 0 | 10.026 | 154.6 | 0.064851229 | 3 | 0 | 0 |
| 1 | 11.299 | 53.4 | 0.21159176 | 4 | 1 | 1 |
| 1 | 47.425 | 37.8 | 1.25462963 | 5 | 1 | 1 |
| 0 | 6.029 | 78.6 | 0.076704835 | 4 | 0 | 0 |
| 1 | 14 | 58.7 | 0.238500852 | 5 | 1 | 1 |
| 1 | 6.686 | 130 | 0.051430769 | 3 | 1 | 1 |
| 0 | 101.073 | 231.6 | 0.436411917 | 3 | 0 | 0 |
| 0 | 6.658 | 75.3 | 0.088419655 | 3 | 0 | 0 |
| 0 | 9.968 | 172 | 0.057953488 | 3 | 0 | 0 |
| 0 | 40.866 | 69.9 | 0.584635193 | 4 | 0 | 0 |
| 0 | 33.098 | 158.5 | 0.208820189 | 3 | 1 | 0 |
| 0 | 6.887 | 83.5 | 0.082479042 | 2 | 1 | 0 |
| 1 | 17 | 34 | 0.5 | 3 | 1 | 1 |
| 0 | 17.547 | 199.7 | 0.0878668 | 3 | 0 | 0 |
| 0 | 20.283 | 117.4 | 0.172768313 | 2 | 1 | 0 |
| 0 | 10.497 | 178.2 | 0.058905724 | 3 | 0 | 0 |
| 1 | 15.949 | 39.9 | 0.399724311 | 5 | 0 | 1 |
| 1 | 35.974 | 54.6 | 0.658864469 | 4 | 1 | 1 |
| 0 | 9.174 | 188.8 | 0.048591102 | 3 | 0 | 0 |
| 0 | 6 | 50.2 | 0.119521912 | 3 | 0 | 0 |
| 0 | 41.1 | 119.3 | 0.34450964 | 2 | 0 | 0 |
| 0 | 8.485 | 85.8 | 0.098892774 | 1 | 1 | 0 |
| 0 | 10.201 | 132.2 | 0.077163389 | 3 | 0 | 1 |
| 0 | 5.131 | 40.3 | 0.127320099 | 2 | 1 | 0 |
| 0 | 8.475 | 151.9 | 0.055793285 | 4 | 1 | 0 |
| 0 | 10.166 | 87.5 | 0.116182857 | 3 | 0 | 0 |
| 0 | 16.535 | 108.6 | 0.152255985 | 3 | 0 | 0 |
| 0 | 9.569 | 113 | 0.084681416 | 3 | 1 | 0 |
| 1 | 100 | 78 | 1.282051282 | 4 | 0 | 1 |
| 0 | 3.297 | 100 | 0.03297 | 2 | 1 | 0 |
| 1 | 10.986 | 132.3 | 0.083038549 | 4 | 1 | 1 |
| 1 | 100 | 103 | 0.970873786 | 5 | 0 | 1 |
| 1 | 25.853 | 66.2 | 0.390528701 | 5 | 0 | 1 |
| 0 | 6.358 | 238 | 0.026714286 | 2 | 1 | 0 |
| 0 | 42.47 | 142 | 0.299084507 | 3 | 0 | 0 |
| 1 | 86.108 | 121 | 0.711636364 | 4 | 1 | 1 |
| 1 | 78.927 | 133 | 0.59343609 | 4 | 1 | 1 |
| 1 | 17.864 | 41.4 | 0.431497585 | 4 | 1 | 1 |
| 1 | 20.467 | 31 | 0.660225806 | 4 | 0 | 1 |
| 1 | 10994 | 74 | 148.5675676 | 5 | 0 | 1 |
| 0 | 19.694 | 120 | 0.164116667 | 3 | 0 | 0 |
| 0 | 12.429 | 201 | 0.061835821 | 2 | 1 | 0 |
| 0 | 13.893 | 120.6 | 0.115199005 | 2 | 0 | 0 |
| 0 | 22.135 | 112 | 0.197633929 | 3 | 0 | 0 |
| 1 | 1.291 | 30 | 0.043033333 | 4 | 1 | 1 |
| 0 | 43.569 | 221 | 0.197144796 | 3 | 0 | 0 |
| 1 | 102.469 | 75 | 1.366253333 | 3 | 1 | 1 |
| 0 | 13.479 | 131 | 0.10289313 | 3 | 0 | 0 |
| 1 | 1892.87 | 76 | 24.90618421 | 4 | 0 | 1 |
| 0 | 15.54 | 115 | 0.135130435 | 3 | 0 | 0 |
| 0 | 23.92 | 50 | 0.4784 | 3 | 0 | 0 |
| 0 | 9.67 | 133.6 | 0.07238024 | 3 | 1 | 0 |
| 0 | 9.384 | 120 | 0.0782 | 3 | 1 | 1 |
| 0 | 7 | 142 | 0.049295775 | 3 | 0 | 0 |
| 0 | 26.105 | 208 | 0.125504808 | 3 | 0 | 0 |
| 1 | 42.169 | 48 | 0.878520833 | 4 | 0 | 1 |
| 0 | 7.185 | 107 | 0.067149533 | 3 | 0 | 1 |
| 0 | 48.306 | 68.5 | 0.70519708 | 3 | 1 | 1 |
| 1 | 21.582 | 84.5 | 0.255408284 | 3 | 1 | 1 |
| 1 | 17.22 | 165 | 0.104363636 | 3 | 1 | 1 |
| 1 | 9.55 | 100 | 0.0955 | 3 | 1 | 1 |
| 0 | 12.997 | 45 | 0.288822222 | 3 | 1 | 1 |
| 0 | 4.872 | 163.6 | 0.029779951 | 3 | 0 | 0 |
| 0 | 10.828 | 140.4 | 0.077122507 | 3 | 1 | 0 |
| 0 | 7.275 | 136 | 0.053492647 | 3 | 1 | 1 |
| 0 | 8.497 | 109 | 0.077954128 | 3 | 0 | 1 |
| 1 | 98.918 | 34.3 | 2.883906706 | 4 | 0 | 1 |
| 1 | 33.269 | 77.2 | 0.430945596 | 4 | 0 | 1 |
| 0 | 16.551 | 77.5 | 0.21356129 | 3 | 1 | 0 |
| 0 | 6.159 | 117 | 0.052641026 | 3 | 0 | 0 |
| 0 | 5.635 | 92 | 0.06125 | 3 | 0 | 0 |
| 1 | 15.344 | 52 | 0.295076923 | 3 | 0 | 1 |
| 0 | 17.656 | 300 | 0.058853333 | 3 | 0 | 0 |
| 0 | 3.559 | 243 | 0.014646091 | 3 | 0 | 0 |
| 0 | 2.344 | 97 | 0.024164948 | 3 | 1 | 1 |
| 1 | 5.473 | 85 | 0.064388235 | 4 | 1 | 1 |
| 0 | 9.527 | 212 | 0.044938679 | 3 | 0 | 0 |
| 0 | 8.33 | 135 | 0.061703704 | 3 | 0 | 0 |
| 1 | 127.57 | 73 | 1.747534247 | 4 | 1 | 1 |
| 1 | 54.787 | 201.6 | 0.271760913 | 5 | 1 | 1 |
| 0 | 35.045 | 133 | 0.263496241 | 2 | 1 | 0 |
| 1 | 48.473 | 194 | 0.249860825 | 5 | 1 | 1 |
| 0 | 3.44 | 79.5 | 0.04327044 | 3 | 0 | 0 |
| 0 | 16.688 | 122.3 | 0.136451349 | 3 | 0 | 0 |
| 0 | 8.359 | 117 | 0.071444444 | 3 | 1 | 0 |
| 1 | 373.89 | 125 | 2.99112 | 5 | 0 | 1 |
| 1 | 228.96 | 56 | 4.088571429 | 5 | 1 | 1 |
| 0 | 7.499 | 84 | 0.08927381 | 2 | 0 | 0 |
| 0 | 17.105 | 268 | 0.063824627 | 3 | 0 | 0 |
| 1 | 136.942 | 170 | 0.805541176 | 5 | 1 | 1 |
| 1 | 666.99 | 222 | 3.004459459 | 5 | 1 | 1 |
| 0 | 16.907 | 116 | 0.14575 | 3 | 1 | 0 |
| 0 | 12.2 | 50 | 0.244 | 3 | 1 | 0 |
| 0 | 8.375 | 130 | 0.064423077 | 3 | 0 | 0 |
| 0 | 28.015 | 160 | 0.17509375 | 3 | 0 | 0 |
| 1 | 360.3 | 66.2 | 5.442598187 | 5 | 0 | 1 |
| 1 | 90.729 | 288 | 0.31503125 | 3 | 0 | 1 |
| 1 | 216.89 | 252 | 0.860674603 | 5 | 1 | 1 |
| 0 | 1.641 | 43 | 0.038162791 | 3 | 0 | 0 |
| 0 | 10.009 | 210 | 0.047661905 | 3 | 1 | 0 |
| 0 | 19.903 | 210 | 0.09477619 | 3 | 0 | 0 |
| 0 | 24.26 | 127 | 0.191023622 | 3 | 0 | 0 |
| 0 | 11.109 | 114 | 0.097447368 | 3 | 0 | 0 |
| 0 | 15.026 | 94.5 | 0.159005291 | 2 | 1 | 0 |
| 1 | 104.825 | 28 | 3.74375 | 5 | 0 | 1 |
| 1 | 50.466 | 59 | 0.855355932 | 4 | 1 | 1 |
| 1 | 11.697 | 27.3 | 0.428461538 | 4 | 1 | 1 |
| 1 | 41.605 | 108 | 0.385231481 | 5 | 0 | 1 |
| 0 | 7.192 | 59 | 0.121898305 | 3 | 1 | 1 |
| 0 | 12.495 | 293 | 0.042645051 | 2 | 0 | 0 |
| 1 | 205.104 | 101 | 2.030732673 | 5 | 1 | 1 |
| 0 | 8.466 | 90 | 0.094066667 | 3 | 0 | 0 |
| 0 | 15.734 | 110 | 0.143036364 | 3 | 1 | 0 |
| 0 | 10.506 | 194 | 0.054154639 | 2 | 0 | 0 |
| 1 | 4.108 | 136 | 0.030205882 | 5 | 1 | 1 |
| 0 | 1.662 | 200 | 0.00831 | 3 | 0 | 0 |
| 0 | 9.973 | 157 | 0.063522293 | 2 | 0 | 0 |
| 0 | 16.018 | 83 | 0.192987952 | 3 | 0 | 0 |
| 1 | 20.702 | 42.3 | 0.489408983 | 4 | 1 | 1 |
| 1 | 13.113 | 48.4 | 0.270929752 | 5 | 0 | 1 |
| 0 | 11.915 | 166 | 0.071777108 | 3 | 0 | 0 |
| 1 | 102.39 | 94.6 | 1.082346723 | 5 | 1 | 1 |
| 0 | 21.612 | 283 | 0.076367491 | 3 | 1 | 0 |
| 1 | 8.056 | 140 | 0.057542857 | 4 | 1 | 1 |
| 1 | 16.297 | 160 | 0.10185625 | 4 | 1 | 1 |
| 0 | 13.809 | 194 | 0.071180412 | 2 | 0 | 0 |
| 0 | 21.43 | 72.3 | 0.296403873 | 2 | 1 | 1 |
| 1 | 35.209 | 120 | 0.293408333 | 5 | 1 | 1 |
| 1 | 16.026 | 73 | 0.219534247 | 4 | 0 | 1 |
| 0 | 9.709 | 91.728 | 0.105845543 | 3 | 0 | 0 |
| 0 | 0.754 | 60 | 0.012566667 | 4 | 0 | 0 |
| 0 | 21.741 | 145.7 | 0.14921757 | 3 | 0 | 0 |
| 0 | 8.038 | 124.8 | 0.064407051 | 2 | 0 | 0 |
| 0 | 13.778 | 150.92 | 0.0912934 | 4 | 0 | 0 |
| 1 | 85.268 | 95.4 | 0.893794549 | 4 | 1 | 1 |
| 0 | 6.016 | 56.16 | 0.107122507 | 3 | 0 | 1 |
| 0 | 17.47 | 108.9 | 0.160422406 | 3 | 1 | 0 |
| 0 | 7.983 | 104.553024 | 0.076353602 | 3 | 1 | 0 |
| 1 | 2869.7 | 80 | 35.87125 | 5 | 1 | 1 |
| 0 | 8.158 | 126.129432 | 0.06467959 | 2 | 1 | 0 |
| 0 | 10.076 | 177.152619 | 0.056877511 | 3 | 0 | 0 |
| 0 | 32.823 | 180.48 | 0.181865027 | 4 | 0 | 0 |
| 0 | 10.367 | 101.92 | 0.101717033 | 3 | 0 | 0 |
| 0 | 10.993 | 121.582 | 0.090416345 | 4 | 0 | 0 |
| 0 | 13.874 | 55.728 | 0.248959231 | 4 | 0 | 1 |
| 0 | 29.987 | 99.84 | 0.300350561 | 2 | 1 | 0 |
| 0 | 148.24 | 95.68 | 1.549331104 | 2 | 0 | 0 |
| 0 | 24.988 | 175.9975 | 0.141979289 | 4 | 1 | 1 |
| 1 | 17 | 83.248 | 0.20420911 | 5 | 1 | 1 |
| 1 | 150.04 | 84.084 | 1.78440607 | 5 | 1 | 1 |
| 0 | 98.65 | 115.2 | 0.856336806 | 3 | 0 | 0 |
| 1 | 1.322 | 75.088 | 0.017606009 | 4 | 0 | 1 |
| 1 | 12.173 | 56 | 0.217375 | 3 | 1 | 1 |
| 0 | 19.018 | 80.631 | 0.235864618 | 3 | 1 | 0 |
| 0 | 19.076 | 198 | 0.096343434 | 3 | 0 | 0 |
| 0 | 18.718 | 153.594 | 0.12186674 | 3 | 0 | 0 |
| 1 | 134.395 | 157.248 | 0.854669058 | 5 | 0 | 1 |
| 1 | 7.022 | 62.04 | 0.113185042 | 3 | 1 | 1 |
| 0 | 20.145 | 63.984 | 0.314844336 | 2 | 0 | 0 |
| 1 | 25.376 | 122.43 | 0.20726946 | 2 | 1 | 1 |
| 0 | 2.508 | 96.237 | 0.026060663 | 3 | 1 | 0 |
| 0 | 11.432 | 72.16 | 0.158425721 | 3 | 0 | 0 |
| 0 | 5.668 | 77.76 | 0.072890947 | 2 | 0 | 0 |
| 0 | 9.929 | 148.029 | 0.067074695 | 2 | 0 | 1 |
| 1 | 86.099 | 193.98 | 0.443855037 | 5 | 1 | 1 |
| 1 | 530.51 | 128.832 | 4.117843393 | 5 | 1 | 1 |
| 1 | 10.546 | 65.6 | 0.160762195 | 2 | 1 | 1 |
| 0 | 8.019 | 122.98 | 0.065205725 | 3 | 1 | 0 |
| 0 | 11.9 | 107.52 | 0.110677083 | 3 | 0 | 0 |
| 0 | 16.863 | 80.784 | 0.20874183 | 3 | 0 | 0 |
| 0 | 4.948 | 116.325 | 0.042535998 | 3 | 0 | 0 |
| 0 | 9.535 | 127.452 | 0.074812478 | 2 | 0 | 0 |
| 0 | 7.403 | 51.156 | 0.144714208 | 4 | 1 | 1 |
| 1 | 92.082 | 60.8 | 1.514506579 | 5 | 1 | 1 |
| 0 | 20.703 | 194.59 | 0.106392929 | 1 | 0 | 0 |
| 1 | 975.28 | 88.2 | 11.05759637 | 4 | 1 | 1 |
| 1 | 5.467 | 34.216 | 0.159779051 | 4 | 1 | 1 |
| 0 | 15.013 | 58.368 | 0.257212856 | 3 | 0 | 0 |
| 1 | 25.161 | 87.822 | 0.286499966 | 5 | 0 | 1 |
| 1 | 68.619 | 158.76 | 0.432218443 | 5 | 0 | 1 |
| 1 | 11.728 | 39.68 | 0.295564516 | 5 | 1 | 1 |
| 1 | 31.429 | 89.01 | 0.353095158 | 5 | 1 | 1 |
| 0 | 85.171 | 137.883 | 0.617704866 | 3 | 1 | 0 |
| 0 | 12.992 | 60.84 | 0.213543721 | 3 | 0 | 0 |
| 1 | 24.417 | 36.96 | 0.660633117 | 5 | 1 | 1 |
| 0 | 5.293 | 100.5525 | 0.052639169 | 4 | 0 | 0 |
| 0 | 38.776 | 102.396 | 0.378686667 | 4 | 1 | 0 |
| 0 | 26.387 | 209.23 | 0.126114802 | 4 | 0 | 0 |
| 1 | 77.149 | 63.878 | 1.207755409 | 5 | 0 | 1 |
| 0 | 6.432 | 103.04 | 0.06242236 | 4 | 0 | 0 |
| 1 | 193.113 | 112.332 | 1.71912723 | 5 | 0 | 1 |
| 0 | 27.943 | 269.742 | 0.10359158 | 4 | 1 | 0 |
| 1 | 20.051 | 62.4 | 0.321330128 | 4 | 1 | 1 |
| 0 | 3.84 | 150.255 | 0.025556554 | 3 | 1 | 0 |
| 0 | 363.82 | 129.168 | 2.816641893 | 5 | 1 | 1 |
| 0 | 5.758 | 61.776 | 0.093207718 | 3 | 0 | 0 |
| 1 | 13.367 | 40.32 | 0.331522817 | 4 | 0 | 1 |
| 1 | 7.342 | 15 | 0.489466667 | 5 | 0 | 1 |
| 1 | 84.453 | 18.4 | 4.589836957 | 5 | 1 | 1 |
| 1 | 6.737 | 49.632 | 0.135739039 | 3 | 1 | 1 |
| 1 | 4.08 | 45.15 | 0.090365449 | 3 | 1 | 1 |
| 1 | 38.961 | 103.88 | 0.375057759 | 4 | 1 | 1 |
| 1 | 7.475 | 69.12 | 0.108145255 | 3 | 1 | 1 |
| 0 | 12.567 | 49.6 | 0.253366935 | 3 | 0 | 0 |
| 0 | 26.785 | 40.5 | 0.661358025 | 3 | 1 | 0 |
| 0 | 13.665 | 217 | 0.06297235 | 3 | 0 | 0 |
| 0 | 10.03 | 147 | 0.068231293 | 4 | 0 | 1 |
| 1 | 7.463 | 89.01 | 0.083844512 | 3 | 1 | 1 |
| 0 | 7.129 | 108.36 | 0.065789959 | 3 | 1 | 0 |
| 1 | 12.218 | 74.518 | 0.163960385 | 4 | 1 | 1 |
| 0 | 11.324 | 106.2 | 0.106629002 | 4 | 1 | 1 |
| 0 | 15.212 | 125.424 | 0.121284603 | 3 | 1 | 1 |
| 1 | 65.18 | 42.77 | 1.523965396 | 5 | 1 | 1 |
| 1 | 100 | 80 | 1.25 | 5 | 1 | 1 |
| 0 | 12.41 | 172.872 | 0.071787218 | 3 | 0 | 0 |
| 0 | 15.738 | 80 | 0.196725 | 3 | 0 | 0 |
| 1 | 36.197 | 68.952 | 0.524959392 | 4 | 1 | 1 |
| 0 | 15.525 | 85.28 | 0.182047373 | 3 | 0 | 0 |
| 0 | 3.094 | 70.56 | 0.043849206 | 3 | 1 | 1 |
| 1 | 38.852 | 49.2 | 0.789674797 | 3 | 1 | 1 |
| 0 | 2.476 | 87.986 | 0.028140841 | 4 | 0 | 0 |
| 0 | 8.62 | 100.862 | 0.085463306 | 3 | 0 | 0 |
| 0 | 8.555 | 75.504 | 0.113305255 | 4 | 0 | 1 |
| 0 | 11.661 | 148.72 | 0.078409091 | 3 | 0 | 0 |
| 0 | 6.572 | 65.016 | 0.10108281 | 4 | 1 | 0 |
| 0 | 39.143 | 127.89 | 0.306067714 | 4 | 1 | 0 |
| 0 | 33.312 | 194.176 | 0.171555702 | 3 | 1 | 0 |
| 0 | 15.914 | 351.288 | 0.045301861 | 4 | 1 | 1 |
| 0 | 60.352 | 182.16 | 0.331313131 | 3 | 1 | 0 |
| 1 | 16.607 | 62.208 | 0.266959234 | 4 | 1 | 1 |
| 0 | 9.794 | 110.448 | 0.088675214 | 4 | 0 | 0 |
| 1 | 27.074 | 93.288 | 0.290219535 | 2 | 1 | 1 |
| 0 | 8.173 | 100.1 | 0.081648352 | 4 | 1 | 0 |
| 1 | 4.345 | 80 | 0.0543125 | 3 | 0 | 1 |
| 0 | 19.431 | 112.8 | 0.172260638 | 3 | 1 | 0 |
| 0 | 29.101 | 126.36 | 0.230302311 | 3 | 0 | 0 |
| 1 | 175.023 | 129.532 | 1.351195071 | 5 | 0 | 1 |
| 1 | 595.72 | 95.58 | 6.232684662 | 5 | 1 | 1 |
| 1 | 595.72 | 61.92 | 9.620801034 | 5 | 1 | 1 |
| 0 | 18.15 | 232.32 | 0.078125 | 4 | 1 | 0 |
| 0 | 7.139 | 47.52 | 0.150231481 | 5 | 0 | 0 |
| 0 | 6.616 | 99.36 | 0.066586151 | 3 | 0 | 0 |
| 0 | 3.054 | 80 | 0.038175 | 3 | 0 | 0 |
| 0 | 5.489 | 111.672 | 0.049152876 | 3 | 0 | 0 |
| 0 | 10.06 | 116.928 | 0.086035851 | 3 | 1 | 1 |
| 1 | 19.979 | 89.505 | 0.22321658 | 3 | 0 | 1 |
| 0 | 1.753 | 39.375 | 0.044520635 | 3 | 1 | 0 |
| 0 | 14.481 | 281.88 | 0.051372925 | 3 | 0 | 0 |
| 0 | 7.019 | 137.592 | 0.05101314 | 5 | 0 | 0 |
| 1 | 1608.098 | 51.772 | 31.06115275 | 5 | 0 | 1 |
| 0 | 62.787 | 139.748 | 0.449287289 | 5 | 1 | 0 |
| 1 | 10.454 | 76.32 | 0.136975891 | 4 | 1 | 1 |
| 0 | 7.192 | 80 | 0.0899 | 3 | 0 | 0 |
| 1 | 128.928 | 103.2 | 1.249302326 | 5 | 0 | 1 |
| 0 | 28.379 | 214.65 | 0.132210575 | 3 | 1 | 1 |
| 0 | 37.542 | 114.48 | 0.32793501 | 3 | 1 | 0 |
| 1 | 226.642 | 120.05 | 1.88789671 | 5 | 1 | 1 |
| 0 | 5.483 | 80 | 0.0685375 | 5 | 1 | 0 |
| 0 | 2.364 | 24 | 0.0985 | 4 | 1 | 0 |
| 0 | 17.033 | 171.808 | 0.099139737 | 3 | 1 | 0 |
| 1 | 6.33 | 24.7 | 0.256275304 | 3 | 0 | 1 |
| 0 | 45.73 | 91.8 | 0.498148148 | 2 | 0 | 0 |
| 0 | 18.204 | 223.44 | 0.081471536 | 2 | 1 | 0 |
| 0 | 6.881 | 110.295 | 0.062387234 | 4 | 1 | 0 |
| 0 | 7.521 | 80 | 0.0940125 | 3 | 1 | 0 |
| 1 | 33.467 | 40.59 | 0.824513427 | 5 | 1 | 1 |
| 1 | 35.932 | 54.12 | 0.663932003 | 3 | 1 | 1 |
| 0 | 8.417 | 202.419 | 0.041582065 | 3 | 0 | 0 |
| 0 | 6.372 | 86.944 | 0.073288554 | 4 | 0 | 1 |
| 1 | 21.46 | 176.384 | 0.121666364 | 3 | 0 | 1 |
| 0 | 12.711 | 143.259 | 0.08872741 | 2 | 0 | 0 |
| 1 | 25.519 | 53.76 | 0.47468378 | 3 | 1 | 1 |
| 0 | 11.764 | 148.23 | 0.079363152 | 2 | 1 | 0 |
| 0 | 9.687 | 140.3 | 0.069044904 | 2 | 1 | 0 |
| 1 | 51.189 | 59.904 | 0.854517228 | 4 | 1 | 1 |
| 0 | 12.156 | 189.54 | 0.06413422 | 3 | 0 | 0 |
| 0 | 7.45 | 64.155 | 0.11612501 | 4 | 0 | 0 |
| 0 | 4.805 | 58.88 | 0.081606658 | 4 | 0 | 0 |
| 1 | 13.607 | 116.229 | 0.117070611 | 3 | 0 | 1 |
| 1 | 20.913 | 69.531 | 0.300772317 | 3 | 1 | 1 |
| 1 | 43.147 | 126.672 | 0.340619869 | 4 | 1 | 1 |
| 0 | 14.281 | 129.25 | 0.110491296 | 3 | 1 | 0 |
| 0 | 17.621 | 134.64 | 0.130874926 | 3 | 0 | 0 |
| 1 | 85.486 | 101.4 | 0.843057199 | 5 | 1 | 1 |
| 1 | 74.646 | 74.448 | 1.002659574 | 5 | 0 | 1 |
| 1 | 14.777 | 25.2 | 0.586388889 | 5 | 1 | 1 |
| 1 | 534.543 | 81.6 | 6.550772059 | 3 | 1 | 1 |
| 0 | 1.715 | 63.492 | 0.027011277 | 2 | 1 | 1 |
| 0 | 17.774 | 507.6 | 0.03501576 | 3 | 0 | 0 |
| 1 | 15.156 | 80.652 | 0.187918465 | 4 | 0 | 1 |
| 0 | 7.635 | 77.4 | 0.098643411 | 4 | 1 | 0 |
| 1 | 8.854 | 63.232 | 0.140024038 | 3 | 0 | 1 |
| 0 | 2.063 | 156.774 | 0.01315907 | 3 | 1 | 0 |
| 0 | 12.59 | 101.844 | 0.123620439 | 4 | 0 | 0 |
| 0 | 9.283 | 124.656 | 0.074468939 | 4 | 0 | 0 |
| 0 | 5.708 | 78.44 | 0.072768995 | 3 | 0 | 0 |
| 1 | 20.442 | 78.4 | 0.260739796 | 3 | 1 | 1 |
| 1 | 41.7 | 111.628 | 0.373562189 | 5 | 1 | 1 |
| 0 | 5.8 | 86 | 0.06744186 | 2 | 0 | 0 |
| 1 | 35.132 | 54.6 | 0.643443223 | 5 | 0 | 1 |
| 0 | 6.294 | 103.88 | 0.060589141 | 4 | 1 | 0 |
| 0 | 11.629 | 252.288 | 0.046094146 | 3 | 1 | 0 |
| 1 | 11.51 | 134.784 | 0.085395893 | 3 | 1 | 1 |
| 0 | 15.181 | 68.31 | 0.222236861 | 3 | 0 | 0 |
| 0 | 10.679 | 127.624 | 0.083675484 | 3 | 0 | 0 |
| 1 | 120.73 | 112.896 | 1.069391298 | 5 | 0 | 1 |
| 1 | 80.165 | 148.77 | 0.538851919 | 4 | 1 | 1 |
| 0 | 8.676 | 51.646 | 0.167989777 | 3 | 0 | 0 |
| 0 | 9.843 | 145.53 | 0.067635539 | 3 | 0 | 0 |
| 0 | 70.525 | 112.896 | 0.62468998 | 3 | 0 | 0 |
| 1 | 7.482 | 80 | 0.093525 | 4 | 1 | 1 |
| 1 | 1584.295 | 92.278 | 17.16871844 | 5 | 1 | 1 |
| 1 | 0.755 | 70.52 | 0.010706183 | 4 | 0 | 1 |
| 0 | 0.974 | 45.12 | 0.021586879 | 3 | 0 | 0 |
| 0 | 8.138 | 100.7 | 0.0808143 | 3 | 0 | 0 |
| 0 | 32.291 | 109.074 | 0.296046721 | 3 | 1 | 0 |
| 0 | 7.015 | 159.6 | 0.043953634 | 3 | 1 | 0 |
| 0 | 9.563 | 39.775 | 0.240427404 | 2 | 1 | 0 |
| 0 | 7.662 | 131.1 | 0.058443936 | 4 | 1 | 1 |
| 0 | 11.112 | 173.46 | 0.064060879 | 3 | 0 | 0 |
| 0 | 21.612 | 164.736 | 0.131191725 | 3 | 1 | 0 |
| 0 | 7.729 | 102.08 | 0.075715125 | 4 | 1 | 0 |
| 0 | 29.975 | 59.976 | 0.499783247 | 3 | 1 | 1 |
| 1 | 18.574 | 43.89 | 0.42319435 | 5 | 0 | 1 |
| 0 | 11.83 | 81.18 | 0.145725548 | 3 | 0 | 0 |
| 1 | 657.443 | 78.144 | 8.413224304 | 5 | 1 | 1 |
| 0 | 6.852 | 37.44 | 0.183012821 | 4 | 1 | 0 |
| 1 | 16.526 | 46.98 | 0.351766709 | 3 | 0 | 1 |
| 0 | 8.973 | 49.98 | 0.179531813 | 4 | 1 | 0 |
| 0 | 6.913 | 114.268 | 0.060498127 | 4 | 0 | 0 |
| 1 | 87.21 | 89.04 | 0.979447439 | 4 | 0 | 1 |
| 0 | 5.281 | 63.18 | 0.083586578 | 4 | 0 | 0 |
| 1 | 18.434 | 89.1 | 0.206891134 | 3 | 0 | 1 |
| 1 | 17.894 | 46.44 | 0.385314384 | 4 | 1 | 1 |
| 0 | 0.835 | 61.318 | 0.013617535 | 3 | 1 | 0 |
| 1 | 15.655 | 62.37 | 0.251002084 | 3 | 1 | 1 |
| 0 | 12.315 | 165.672 | 0.074333623 | 3 | 1 | 0 |
| 1 | 14.363 | 61.776 | 0.232501295 | 5 | 1 | 1 |
| 1 | 33.049 | 142.56 | 0.231825196 | 4 | 1 | 1 |
| 1 | 369.9 | 54.74 | 6.757398612 | 4 | 1 | 1 |
| 0 | 6.635 | 130.331 | 0.05090884 | 2 | 0 | 0 |
| 0 | 7.087 | 135.68 | 0.052233196 | 4 | 0 | 0 |
| 0 | 13.301 | 143.64 | 0.092599554 | 4 | 0 | 0 |
| 1 | 42.826 | 66.6 | 0.643033033 | 4 | 1 | 1 |
| 0 | 12.151 | 26.244 | 0.463001067 | 4 | 1 | 0 |
| 0 | 20.554 | 94.668 | 0.21711666 | 3 | 0 | 0 |
| 0 | 37.619 | 133.056 | 0.28273058 | 3 | 0 | 0 |
| 0 | 6.087 | 79.765 | 0.076311666 | 3 | 0 | 0 |
| 1 | 96.348 | 154.752 | 0.622596154 | 5 | 0 | 1 |
| 0 | 8.788 | 84.588 | 0.103891805 | 4 | 0 | 0 |
| 0 | 5.925 | 145.7 | 0.040665752 | 3 | 0 | 0 |
| 1 | 358.539 | 145.7 | 2.46080302 | 5 | 0 | 1 |
| 0 | 17.09 | 99.36 | 0.172000805 | 3 | 1 | 0 |
| 1 | 310.504 | 146.64 | 2.11745772 | 5 | 1 | 1 |
| 1 | 59.309 | 55.2 | 1.074438406 | 4 | 1 | 1 |
| 0 | 11.259 | 141.642 | 0.079489135 | 3 | 1 | 0 |
| 0 | 7.766 | 131.58 | 0.059021128 | 4 | 1 | 1 |
| 1 | 42.94 | 131.1 | 0.327536232 | 5 | 0 | 1 |
| 1 | 189.144 | 136.884 | 1.381783116 | 5 | 0 | 1 |
| 0 | 5.85 | 125.216 | 0.046719269 | 3 | 1 | 0 |
| 0 | 7.836 | 148.23 | 0.052863793 | 3 | 0 | 0 |
| 1 | 11.164 | 60 | 0.186066667 | 4 | 1 | 1 |
| 0 | 8.516 | 80.84 | 0.105343889 | 3 | 0 | 0 |
| 0 | 25.97 | 151.524 | 0.171391991 | 3 | 0 | 0 |
| 1 | 33.135 | 227.15 | 0.145872771 | 4 | 0 | 1 |
| 0 | 4.556 | 46.332 | 0.098333765 | 4 | 1 | 0 |
| 0 | 7.731 | 96 | 0.08053125 | 2 | 1 | 1 |
| 0 | 12.394 | 80 | 0.154925 | 2 | 1 | 1 |
| 0 | 11.882 | 157.248 | 0.075562169 | 3 | 1 | 0 |
| 0 | 8.48 | 90.895 | 0.093294461 | 4 | 1 | 1 |
| 0 | 7.772 | 112.36 | 0.069170523 | 4 | 1 | 0 |
| 0 | 9.305 | 63.6 | 0.146305031 | 4 | 0 | 0 |
| 0 | 11.647 | 80 | 0.1455875 | 3 | 0 | 0 |
| 0 | 1.185 | 64.35 | 0.018414918 | 2 | 0 | 0 |
| 0 | 11.315 | 119.515 | 0.094674309 | 3 | 0 | 0 |
| 1 | 22.487 | 47.12 | 0.477228353 | 3 | 0 | 1 |
| 1 | 421.43 | 95.202 | 4.426692717 | 3 | 0 | 1 |
| 0 | 15.072 | 171.12 | 0.088078541 | 2 | 1 | 0 |
| 0 | 6.061 | 58.28 | 0.103997941 | 4 | 1 | 1 |
| 0 | 7.4 | 185.136 | 0.039970616 | 3 | 1 | 1 |
| 0 | 27.881 | 320.625 | 0.086958285 | 4 | 1 | 0 |
| 0 | 5.286 | 105.78 | 0.049971639 | 4 | 1 | 1 |
| 0 | 1.158 | 123.648 | 0.009365295 | 4 | 1 | 1 |
| 0 | 20.926 | 56.832 | 0.368208052 | 3 | 1 | 0 |
| 0 | 7.667 | 79.288 | 0.096698113 | 2 | 0 | 0 |
| 1 | 179.041 | 125.396 | 1.427804715 | 4 | 0 | 1 |
| 1 | 75.101 | 72.45 | 1.036590752 | 4 | 0 | 1 |
| 0 | 36.761 | 185.318 | 0.198367131 | 3 | 1 | 0 |
| 0 | 11.126 | 80 | 0.139075 | 2 | 1 | 0 |
| 0 | 2.832 | 99.76 | 0.028388132 | 4 | 1 | 0 |
| 0 | 16.756 | 117.6 | 0.142482993 | 4 | 0 | 0 |
| 1 | 32.215 | 105.3 | 0.305935423 | 3 | 0 | 1 |
| 1 | 21.731 | 76.32 | 0.284735325 | 4 | 0 | 1 |
| 0 | 9.785 | 50.995 | 0.191881557 | 3 | 0 | 0 |
| 0 | 12.077 | 121.52 | 0.099382818 | 3 | 0 | 0 |
| 0 | 9.823 | 143 | 0.068692308 | 3 | 0 | 0 |
| 0 | 24.81 | 132.44 | 0.187330112 | 4 | 0 | 0 |
| 0 | 7.953 | 72.8 | 0.109244505 | 4 | 1 | 0 |
| 1 | 10.686 | 46.8 | 0.228333333 | 2 | 1 | 1 |
| 1 | 44.532 | 49.068 | 0.90755686 | 4 | 1 | 1 |
| 0 | 7.104 | 116.16 | 0.061157025 | 4 | 1 | 0 |
| 0 | 36.847 | 181.366 | 0.203163768 | 5 | 0 | 0 |
| 0 | 5.836 | 176.7 | 0.033027731 | 3 | 0 | 0 |
| 0 | 6.144 | 45.696 | 0.134453782 | 2 | 0 | 0 |
| 1 | 21 | 70.312 | 0.298668791 | 4 | 0 | 1 |
| 0 | 9.423 | 198.588 | 0.047449997 | 4 | 0 | 0 |
| 0 | 16.487 | 74 | 0.222797297 | 2 | 1 | 0 |
| 1 | 11.928 | 105.165 | 0.113421766 | 3 | 1 | 1 |
| 1 | 69.92 | 73.8 | 0.947425474 | 4 | 1 | 1 |
| 1 | 48.975 | 110.124 | 0.444725945 | 5 | 1 | 1 |
| 0 | 7.663 | 124.2 | 0.061698873 | 4 | 1 | 0 |
| 0 | 8.612 | 218.24 | 0.039461144 | 4 | 1 | 1 |
| 0 | 4.752 | 84.968 | 0.055926937 | 3 | 1 | 0 |
| 0 | 22.628 | 167.58 | 0.135028046 | 3 | 1 | 0 |
| 1 | 5.417 | 63.591 | 0.08518501 | 3 | 1 | 1 |
| 0 | 10.934 | 103.488 | 0.105654762 | 3 | 1 | 0 |
| 0 | 4.532 | 36.801 | 0.123148827 | 3 | 0 | 1 |
| 0 | 14.941 | 115.92 | 0.128890614 | 3 | 0 | 0 |
| 1 | 6.592 | 35.264 | 0.186932849 | 4 | 0 | 1 |
| 0 | 5.699 | 105.78 | 0.053875969 | 3 | 1 | 0 |
| 0 | 8.96 | 235.41 | 0.038061255 | 3 | 0 | 0 |
| 0 | 11.191 | 64.98 | 0.172222222 | 2 | 0 | 0 |
| 0 | 3.72 | 100.8 | 0.036904762 | 2 | 0 | 0 |
| 0 | 10 | 123.48 | 0.080984775 | 3 | 0 | 1 |
| 0 | 8.146 | 77.4 | 0.105245478 | 4 | 0 | 1 |
| 0 | 11.074 | 56.55 | 0.195826702 | 2 | 0 | 0 |
| 0 | 9.309 | 121.9 | 0.076365874 | 3 | 0 | 0 |
| 1 | 16.915 | 41.16 | 0.41095724 | 4 | 0 | 1 |
| 0 | 13.806 | 122.496 | 0.112705721 | 4 | 1 | 1 |
| 1 | 15.05 | 44.608 | 0.337383429 | 3 | 1 | 1 |
| 1 | 68.151 | 58.52 | 1.164576213 | 3 | 1 | 1 |
| 0 | 6 | 92.4 | 0.064935065 | 4 | 1 | 0 |
| 1 | 18.624 | 82.08 | 0.226900585 | 4 | 1 | 1 |
| 0 | 7.608 | 119.25 | 0.063798742 | 3 | 1 | 0 |
| 1 | 15.645 | 65.892 | 0.237433983 | 4 | 1 | 1 |
| 0 | 6.517 | 136.29 | 0.047817155 | 3 | 1 | 0 |
| 0 | 11.582 | 58.968 | 0.196411613 | 2 | 1 | 0 |
| 0 | 23.748 | 201.096 | 0.118092851 | 3 | 1 | 1 |
| 0 | 9.892 | 154.698 | 0.063943942 | 3 | 0 | 0 |
| 0 | 24.201 | 141.12 | 0.171492347 | 4 | 0 | 0 |
| 0 | 11.693 | 156.6 | 0.074667944 | 3 | 0 | 0 |
| 0 | 6.033 | 66.792 | 0.090325189 | 1 | 0 | 0 |
| 0 | 12.588 | 92 | 0.136826087 | 2 | 0 | 0 |
| 1 | 12.4 | 102.9 | 0.120505345 | 3 | 0 | 1 |
| 1 | 5.073 | 56.84 | 0.089250528 | 3 | 0 | 1 |
| 1 | 404.42 | 50.616 | 7.989963648 | 5 | 1 | 1 |
| 0 | 14.094 | 60 | 0.2349 | 4 | 0 | 1 |
| 1 | 2299.794 | 78.72 | 29.2148628 | 5 | 1 | 1 |
| 0 | 5.665 | 102.396 | 0.055324427 | 2 | 0 | 0 |
| 0 | 6.465 | 105.792 | 0.061110481 | 4 | 1 | 0 |
| 0 | 10.358 | 119.952 | 0.086351207 | 3 | 1 | 0 |
